# Influenza A(H5N1) Virus Resilience in Milk after Thermal Inactivation

**DOI:** 10.3201/eid3011.240772

**Published:** 2024-11

**Authors:** C. Joaquin Caceres, L. Claire Gay, Flavio Cargnin Faccin, Dikshya Regmi, Roberto Palomares, Daniel R. Perez

**Affiliations:** University of Georgia College of Veterinary Medicine, Athens, Georgia, USA

**Keywords:** influenza, food safety, viruses, respiratory infections, zoonoses, Pasteurization, milk, United States

## Abstract

Highly pathogenic avian influenza A(H5N1) detected in dairy cows raises concerns about milk safety. The effects of pasteurization-like temperatures on influenza viruses in retail and unpasteurized milk revealed virus resilience under certain conditions. Although pasteurization contributes to viral inactivation, influenza A virus, regardless of strain, displayed remarkable stability in pasteurized milk.

Recent detection of highly pathogenic avian influenza A(H5N1) virus in US dairy cows raises serious public health concerns (*1*–*3*). Pasteurization, a common process for ensuring milk safety, involves heating milk to specific temperatures for specific lengths of time to eliminate disease-causing bacteria. The most common methods in the United States (*4*) are low-temperature long-time (LTLT, 63°C for 30 minutes) and high-temperature short-time (HTST, 72°C for 15–20 seconds) pasteurization. Recent studies have shown that unpasteurized milk from H5N1-infected cows contains enough virus to infect susceptible animals (*5*). 

We examined pasteurizing milk at various temperatures to evaluate how temperature affects virus viability (Figure 1). It is crucial to emphasize that we do not assert that those conditions in a test tube setting simulate the actual pasteurization process. We used 4 influenza virus strains in this study: 1 laboratory-adapted strain (PR8) and 3 H5N1 strains (Figures 1, 2; Appendix). We spiked commercially available pasteurized whole milk (3.25% fat) with virus strains at a concentration of 10^8^ 50% tissue culture infectious dose/mL of milk or Opti-MEM control media (Fisher Scientific, https://www.fishersci.com). We subjected varying sample volumes (200 μL, 20 μL, and 2 μL) to 3 distinct heat treatments: 63°C for 30 minutes, 72°C for 20 seconds, and 91°C for 20 seconds. In addition, we tested the PR8 strain in both pasteurized and unpasteurized milk to investigate the effect of preheating milk at 37°C for 1 minute before subjecting it to HTST-like conditions. After treatment, we adjusted samples to a final volume of 200 μL and titrated (*6*). 

**Figure 1:**
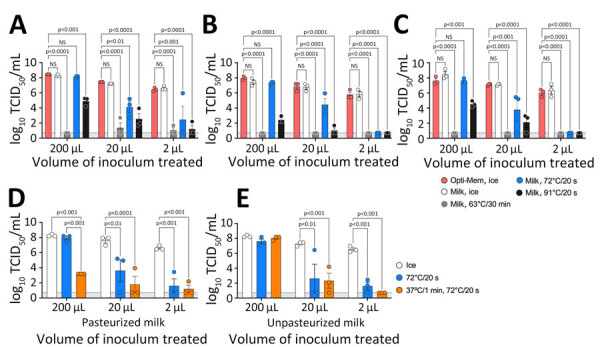
Heat treatment of influenza virus in milk. A–C) We diluted influenza A viruses in Opti-Mem control media (Fisher Scientific, https://www.fishersci.com) or commercial off-the-shelf pasteurized whole milk and heat-treated samples of different volumes at the times and temperatures shown; we calculated time from the moment the sample was placed in the heat block. A sandwich design in a heat block ensured uniform temperature exposure. After treatment, we chilled samples on ice for 5 minutes, adjusted them to a final volume of 200 μL, and titrated by TCID_50_ in MDCK cells (*10*). Results are shown for reverse genetics wild-type strain A/Puerto Rico/8/1934 (H1N1) (A); Vietnam/1203/04, a reverse genetics virus carrying the H5 hemagglutinin and N1 neuraminidase segments from A/Vietnam/1203/2004 (H5N1) in the background of PR/8/34, with the H5 segment modified with a monobasic cleavage site (Δ) (B); and a field isolate of the wild-type highly pathogenic strain A/turkey/Indiana/3707-003/2022 (H5N1) (C). D, E) A/Puerto Rico/8/1934 (H1N1) strain was spiked on pasteurized (D) and unpasteurized (E) milk samples at the times and temperatures shown. Circles indicate individual measurements; error bars indicate 95% CIs. Light gray shaded area indicates log_10_ TCID_50_ value of 1. NS, not significant; TCID_50_, 50% tissue culture infectious dose.

**Figure 2:**
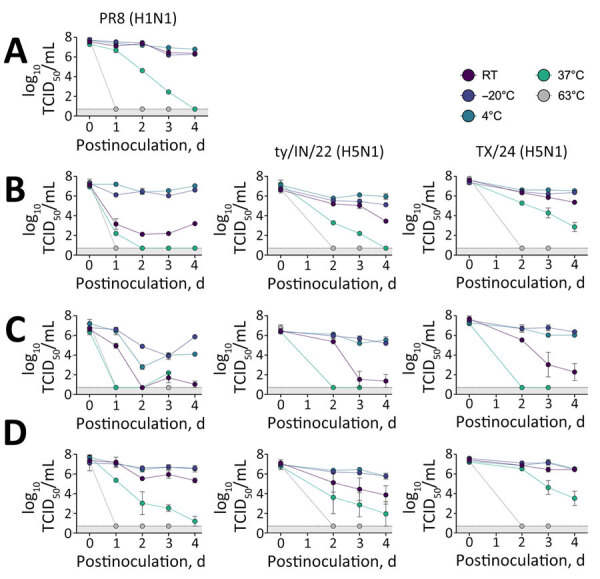
Stability of influenza A in retail and unpasteurized milk. We diluted influenza A viruses in either Opti-Mem control media (Fisher Scientific, https://www.fishersci.com) (A), retail off-the-shelf pasteurized whole milk (B), or 2 different sources of unpasteurized milk: colostrum milk (C) or mature milk (D). We then incubated 200-μL samples for several days at various temperatures, as shown. We subsequently titrated samples by TCID_50_ in MDCK cells. We tested 3 strains: PR8 (H1N1), ty/IN/22 (H5N1), and the reverse genetics version of TX/24 (H5N1). Unpasteurized colostrum milk produced during the first few days after birth contains high levels of immunoglobulins and antimicrobial peptides that might have had an effect in decreasing virus survival. PR8 (H1N1), wild-type strain A/Puerto Rico/8/1934 (H1N1); RT, room temperature; TCID_50_, 50% tissue culture infectious dose; TX/24, wild-type strain A/Texas/37/2024 (H5N1); ty/IN/22, wild-type highly pathogenic strain A/turkey/Indiana/3707-003/2022 (H5N1).

We observed no significant (i.e., p<0.05) difference in viral titer between influenza viruses diluted in control media and in milk. All 3 viruses tested in this assay (PR8, VN/04 ΔH5N1, and ty/IN/22) behaved similarly (Figure 1, panels A–C). Heat treatment at 63°C for 30 minutes effectively reduced viral viability below the limit of detection. For samples treated at 72°C for 20 seconds, titer reduction was inversely proportional to sample volume, with a nonsignificant decrease observed in 200-μL samples. Conversely, we observed significant (p<0.05) titer reduction in 20-μL and 2-μL samples at 72°C. Treatment at 91°C for 20 seconds also resulted in significant titer reduction inversely proportional to sample volume. Preheating samples to 37°C for 1 minute before beginning HTST (Figure 1, panels D, E) accelerated virus inactivation and was more pronounced in smaller volumes of milk (Figure 1, panel A). 

To investigate how different types of milk and storage temperatures affected the stability of influenza virus strains (Figure 2, panel B), we tested pasteurized milk, unpasteurized colostrum milk (Figure 2, panel C), and unpasteurized mature milk (Figure 2, panel D). We stored the milk samples spiked with viruses at different temperatures for up to 4 days. We included a control sample in virus media for comparison. Those viruses showed remarkable resilience in unpasteurized milk, remaining infectious for >4 days at temperatures other than 63°C, at which temperature virus was inactivated within 24 hours. Unpasteurized colostrum milk showed increased virus inactivation, perhaps because of the presence of immunoglobulins. 

Although our study offers valuable insights, it is critical to note that spiking viruses into milk might not perfectly mimic a natural infection. However, influenza viruses, being enveloped, are generally less stable than the nonenveloped viruses used in previous studies, showcasing this limitation (*7*). 

Commercially available milk undergoes pasteurization and homogenization processes. Whereas H5N1 vRNA has been detected in some store-bought milk, the consistent absence of viable virus suggests the pasteurization and homogenization processes might contribute to viral inactivation. Several studies have investigated temperature conditions that mimic HTST pasteurization, with conflicting results. One study (*5*) observed complete virus inactivation only when samples were heated in a PCR machine with the lid on at 105°C but not at 72°C when the lid was replaced with a heat block. That observation aligns with our findings. Another study (*8*) demonstrated complete H5N1 inactivation in spiked milk samples treated under HTST conditions using a thermomixer; however, the timer was initiated only after the samples reached the target temperature (≈58 seconds later), potentially influencing the results. There is compelling evidence of virus inactivation under real-life HTST conditions, suggesting it can effectively lead to complete virus inactivation (*9*). That study estimated that standard US continuous flow HTST parameters would inactivate a significantly higher viral load than typically detected in unpasteurized milk, suggesting the milk supply is likely safe. However, caution is warranted because the industry lacks mandatory testing for H5N1 in milk. Definitively ruling out the presence of live virus might require multiple blind passages in eggs, as is standard procedure in surveillance studies. Our results with the laboratory-adapted PR8 strain are significant in this context, because PR8-spiked milk could serve as an ideal surrogate for testing under commercial pasteurization conditions. Our research, along with understanding factors influencing virus survival in milk, will inform targeted interventions to enhance milk safety and reassure consumers regarding emerging viral threats. 

Appendix Methods and results from study of thermal inactivation of influenza A virus in dairy milk.
