# Supplementary material for: Influenza A(H5N1) Virus Resilience in Milk after Thermal Inactivation
Source: Emerg Infect Dis. 2024 Nov;30(11):2426–9. doi: 10.3201/eid3011.240772 (PMC11521188; doi:10.3201/eid3011.240772)
Supplement: Appendix — Methods and results from study of thermal inactivation of influenza A virus in dairy milk. [file 24-0772-Techapp-s1.pdf]

Article DOI: https://doi.org/10.3201/eid3011.260772
EID cannot ensure accessibility for supplementary materials supplied by authors. Readers who have difficulty accessing supplementary content should contact the authors for assistance.

Influenza A(H5N1) Virus Resilience in Milk after Thermal Inactivation

Appendix

Materials and Methods

Cells and Viruses

Madin-Darby canine kidney (MDCK) was a kind gift from Robert Webster of St. Jude's Childrens Research Hospital. Cells were maintained in Sigma-Aldrich Dulbecco's Modified Eagles Medium, containing 10% fetal bovine serum, 1% antibiotic/antimycotic solution(AB,) and 1% L-Glutamine (https://www.sigmaaldrich.com). Cells were cultured at 37ºC under 5% CO2. The wild-type strain A/turkey/Indiana/3707-003/2022 (H5N1) HPAIV (ty/IN/22) was kindly provided by Darrell Kapczynski of Southeast Poultry Research Laboratory, US National Poultry Research Center, Agricultural Research Service, USDA. Laboratory-adapted A/Puerto Rico/8/34 (H1N1) (PR8) and a low pathogenic avian influenza virus (LPAIV) with H5N1 surface proteins (A/Vietnam/04/2003, VN/04 ΔH5N1) were obtained by reverse genetics and have been previously described (*1*). The RG-plasmids for wild-type A/Texas/37/2024 (H5N1) (TX/24) were designed in the lab and obtained from Twist Biosciences (https://www.twistbioscience.com). Whole plasmid sequence integrity was verified independently by Plasmidsaurus (https://www.plasmidsaurus.com) using Oxford Nanopore technology (https://nanoporetech.com) with custom analysis and annotation. Viral stocks were prepared in 10-day old SPF embryonated eggs. Viral sequences were confirmed in house by next-generation sequencing using MiSeq (https://www.illumina.com) as previously described (*2*) and by Sanger sequencing (https://www.psomagen.com).

Thermal Inactivation of Influenza A Viruses in Milk

Thermal stability of PR8, VN/04 ΔH5N1, and ty/IN/22 was evaluated in both Opti-Mem control media and commercially available pasteurized whole milk (3.25% fat, Grade A). Viruses were diluted 1:10 in the respective media, and varying volumes (200 µL, 20 µL, and 2 µL, each in triplicate) were aliquoted into 0.2 mL polypropylene PCR tubes. Samples were maintained on ice until heat treatment, which was conducted in a Sigma-Aldrich Corning LSE digital dry bath equipped with aluminum alloy heating blocks. The heat block was preheated to the target temperature (either 63ºC, 72ºC, or 91ºC) prior to sample transfer. Following sample addition, a second preheated aluminum block was placed atop the tubes to ensure uniform heat distribution, and the incubation timer was initiated (30 min at 63ºC, 20 seconds at either 72ºC or 91ºC). Post-incubation, samples were immediately chilled on ice for 5 minutes, adjusted to a final volume of 200 µL. Viral infectivity was determined using $TCID_{50}$ assays (*3*), with each replicate evaluated in triplicate. For example, for the three 200 µL aliquots processed at a given temperature, a total of 9 independent $TCID_{50}$ assessments were conducted. The $TCID_{50}$ assays were performed in MDCK cells seeded at a density of 1.5 x 10^4 cells per well in 96-well plates 24 hours prior to inoculation. The presence of virus in tissue culture supernatant was established by measuring levels of hemagglutination units (HAU) in hemagglutination assays using 50 µL of infected-cell supernatant and an equal volume of 0.5% chicken red blood cells (*3*).

Stability of Influenza A in Retail and Unpasteurized Raw Milk

The thermal stability of PR8, VN/04 ΔH5N1, and TX/24 (H5N1) was investigated in various media: Opti-Mem (control for PR8), commercially available pasteurized whole milk (3.25% fat, Grade A), and two distinct unpasteurized milk samples (designated E and J). Milk E represents colostrum milk collected within the first 7 days postpartum, while Milk J refers to mature milk collected at a later stage of lactation. It is important to note that Milk E and Milk J were sourced from different cows. Moreover, multiple batches of both Milk E and Milk J were obtained as different viruses were tested at different time points and biocontainment conditions.

Viruses were diluted 1:10 in their respective media, and baseline samples (Time 0) were collected prior to exposure to a range of temperatures (–20°C, 4°C, room temperature, 37°C, and 63°C). Samples were collected once daily for four days and stored at –80°C until further analysis. Residual infectious virus titers were determined using the $TCID_{50}$ assay

Graphs and Statistical Analysis

All data analyses and graphs were performed using GraphPad Prism software version 10.3.0 (https://www.graphpad.com). One-way and/or 2-way ANOVA with multiple comparisons were performed; $p<0.05$ was considered significant. Half-life analyses were performed using one-phase decay.

Results

Appendix Table. Half-life (hours) of different influenza strains in different milk samples

		Milk type		
Virus	Temperature, C°	Pasteurized whole (3.25% fat) Grade A	Unpasteurized colostrum	Unpasteurized mature
PR8 (H1N1)	–20	>96	>96	>96
	4	>96	69.4	>96
	RT	31.7	23	>96
	37	10.9	1.2	32.7
	63	<1	<1	<1
ty/IN/22	–20	>96	>96	>96
	4	>96	>96	>96
	RT	>96	44.1	93.1
	37	30.8	2.1	45.5
	63	<1	<1	<1
TX/24	–20	>96	>96	>96
	4	>96	>96	>96
	RT	>96	52.6	>96
	37	69.5	2.1	95.9
	63	<1	<1	<1

References

1. Song H, Nieto GR, Perez DR. A new generation of modified live-attenuated avian influenza viruses using a two-strategy combination as potential vaccine candidates. J Virol. 2007;81:9238–48. PubMed https://doi.org/10.1128/JVI.00893-07

2. Ferreri LM, Ortiz L, Geiger G, Barriga GP, Poulson R, Gonzalez-Reiche AS, et al. Improved detection of influenza A virus from blue-winged teals by sequencing directly from swab material. Ecol Evol. 2019;9:6534–46. PubMed https://doi.org/10.1002/ece3.5232

3. World Health Organization, Global Influenza Program, Global Influenza Surveillance and Response System. Manual for the laboratory diagnosis and virological surveillance of influenza: 2011 [cited 2024 May 20]. https://www.who.int/publications/i/item/manual-for-the-laboratory-diagnosis-and-virological-surveillance-of-influenza
